# Suramin is a potent inhibitor of Chikungunya and Ebola virus cell entry

**DOI:** 10.1186/s12985-016-0607-2

**Published:** 2016-08-31

**Authors:** Lisa Henß, Simon Beck, Tatjana Weidner, Nadine Biedenkopf, Katja Sliva, Christopher Weber, Stephan Becker, Barbara S. Schnierle

**Affiliations:** 1 Department of Virology, Paul-Ehrlich-Institut, Paul-Ehrlich Strasse 51-59, 63225 Langen, Germany; 2 Institute of Virology, Philipps-University Marburg, Hans-Meerwein-Str. 2, 35043 Marburg, Germany; 3 German Center for Infection Research (DZIF) at the Philipps University Marburg, partner site, Gießen-Marburg-Langen, Germany

**Keywords:** Chikungunya virus, Ebola virus, Suramin

## Abstract

**Background:**

Chikungunya virus (CHIKV) is a mosquito-transmitted alphavirus that causes high fever, rash, and recurrent arthritis in humans. It has efficiently adapted to *Aedes albopictus,* which also inhabits temperate regions and currently causes large outbreaks in the Caribbean and Latin America. Ebola virus (EBOV) is a member of the filovirus family. It causes the Ebola virus disease (EDV), formerly known as Ebola hemorrhagic fever in humans and has a mortality rate of up to 70 %. The last outbreak in Western Africa was the largest in history and has caused approximately 25,000 cases and 10,000 deaths. For both viral infections no specific treatment or licensed vaccine is currently available. The bis-hexasulfonated naphthylurea, suramin, is used as a treatment for trypanosome-caused African river blindness. As a competitive inhibitor of heparin, suramin has been described to have anti-viral activity.

**Methods:**

We tested the activity of suramin during CHIKV or Ebola virus infection, using CHIKV and Ebola envelope glycoprotein pseudotyped lentiviral vectors and wild-type CHIKV and Ebola virus.

**Results:**

Suramin efficiently inhibited CHIKV and Ebola envelope-mediated gene transfer while vesicular stomatitis virus G protein pseudotyped vectors were only marginally affected. In addition, suramin was able to inhibit wild-type CHIKV and Ebola virus replication in vitro. Inhibition occurred at early time points during CHIKV infection.

**Conclusion:**

Suramin, also known as Germanin or Bayer-205, is a market-authorized drug, however shows significant side effects, which probably prevents its use as a CHIKV drug, but due to the high lethality of Ebola virus infections, suramin might be valuable against Ebola infections.

## Background

The Chikungunya virus (CHIKV) is a mosquito-transmitted alphavirus that causes flu-like symptoms and arthritis. In about 30 % of cases arthritis can last for months or even years, which may cause substantial economic losses [1, 2]. The virus currently spreads from Africa and the Indian Ocean to the Caribbean and Latin America and is now responsible for large, still-ongoing outbreaks with 1.7 million suspected cases as of October 2014 (www.cdc.gov). The mortality rate is very low (0.1 %), but the infection rates are high (about 30 %) and asymptomatic cases are rare (about 15 %) [3]. Due to climate change, globalization, and vector switching, the virus will most likely continue to cause new, worldwide outbreaks also in more temperate regions like Europe or the USA [4, 5].

In contrast, Ebola virus (EBOV) belongs to the filovirus family and is a single (–)-stranded RNA enveloped virus. EBOV infections cause Ebola hemorrhagic fever in humans with mortality rates of up to 70 %. The last outbreak of EBOV in Western Africa was by far the largest outbreak in history. As of May 12^th^, 2016 an estimated number of 28,616 cases and 11.310 deaths have been reported (www.CDC.gov). For both viral infections no treatment or licensed vaccine exists [2, 6, 7].

CHIKV, like other alphaviruses, enters cells by receptor-mediated endocytosis and a subsequent pH-dependent fusion step. CHIKV has two surface proteins that mediate cell entry: the transmembrane glycoproteins E2 and E1. E2 mediates cell attachment and E1 is a class II viral fusion protein [8]. EBOV cell entry is mediated by a single envelope GP that contains the receptor-binding domain and additionally acts as a fusion protein [9, 10]. Filoviruses utilize a combination of attachment and receptor molecules to enter cells, resulting in a very broad host range. Many factors have been described to interact with EBOV, including proteoglycans. After cell binding, EBOV is taken up by macropinocytosis, followed by low pH-dependent cathepsin B/L-mediated proteolytic cleavage of GP. Finally, binding of the GP to the Niemann-Pick C1 (NPC1) protein as an intracellular receptor is required for the fusion process and virus entry into the cytoplasm [9, 10].

The early steps of viral infections are carried out by the viral glycoproteins which mediate attachment and entry of the virus into the target cell. A tool to investigate the glycoproteins of viruses is the pseudotyping of lentiviral vectors with the desired glycoproteins (here: CHIKV or EBOV) [11]. With this strategy, the lentiviral vectors incorporate a heterologous viral glycoprotein (GP) and thereby acquire the host range of the virus from which the glycoprotein is derived. These pseudotyped vectors enable studies to be carried out without the need for using the native virus, which for Ebola virus reduces the required safety level from the highest level 4 to level 2. We have previously established a chikungunya virus (CHIKV) neutralization assay in a 384-well format [12] and adjusted this multiplex assay for Ebola virus, allowing the analysis of inhibitors of infection in a short period of time, which in cases of virus outbreaks could be of decisive advantage.

Proteoglycans are widely distributed molecules on the cell surface, consisting of a transmembrane protein linked to sulfated glycosaminoglycans (GAGs). Therefore, many pathogens exploit GAGs to cross the cell membrane barrier, using them for initial cell attachment or as entry receptors. These pathogens include several bacteria, parasites, and viruses [13, 14]. It has been shown previously that although CHIKV and Ebola virus enter cells by different pathways, they both use GAGs for their attachment to target cells. Soluble GAGs are able to inhibit pseudotyped vector entry [15–17]. As a competitive inhibitor of GAGs and heparin, suramin has been shown to have anti-viral activity. Suramin, also known as Germanin or Bayer-205, is a market-authorized drug for the treatment of trypanosome-caused river blindness. It has a negative charge and binds to basic side chains of proteins. Several viruses have been described to be inhibited by suramin among them HIV [18, 19] HSV-1 [20], HBV [21], HCV [22], dengue virus [23], EV71 [24], Rift Valley Fever Virus [25] and recently also CHIKV [26, 27]. Here, we extended these observations for CHIKV and EBOV by using pseudotyped-lentiviral vectors carrying envelope glycoproteins and using the wild-type viruses.

## Methods

### Cell culture

All cells used in this study were cultured at 37 °C under 5 % CO_2_. HEK 293 T (CRL-1573), MCF7, and Huh7 (CCL-185) cells were grown in Dulbecco’s modified Eagle medium (DMEM; Lonza, Verviers, Belgium). All media were supplemented with 10 % FCS (v/v; PAA, Pasching, Austria), penicillin (50 units/mL), streptomycin (50 μg/mL), and 5 % L-glutamine (200 mM; Lonza, Verviers, Belgium). Suramin was purchased by Sigma-Aldrich (Munich, German) with a purity ≥99 %.

### Plasmids and DNA

The gene for the CHIKV E3-E1 envelope polyprotein was expressed from plasmid pIRES2-eGFP-CHIKV E3-E1 [12] and the Zaire Ebola virus envelope polyprotein (pEBOV-GP) gene was obtained from C. Goffinet [28]. Furthermore, the plasmids pMDLg/pRRE, pRSVrev, pRRLsinCMV-GFPpre [29], pCSII-Luc [30] (kind gift of N. Somia) and pHIT-G (encoding VSV-G; [31]) were used for the production of vector particles.

### Lentiviral vector particle production

HEK 293 T cells were seeded in 10 cm dishes in 10 ml DMEM. After 16 h, subconfluent cells (~80 % density) were cotransfected with the plasmids pRRLsinCMV-GFPpre or pCSII-Luc (10 μg), pMDLg/pRRE (6.5 μg), pRSVrev (2.5 μg), and pHIT-G, pEBOV-GP (3.5 μg) or pIRES2-eGFP-CHIKV E3-E1 (5.3 μg) using Lipofectamine 2000 (according to the manufacturer’s protocol; Life Technologies). After 24 h of incubation, the medium was replaced with 5 ml fresh DMEM per dish. Another 24 h later, the supernatant containing vector particles was harvested, sterile filtered with 0.45 μm filters (Sartorius, Göttingen, Germany) and frozen at –80 °C.

### Transduction of cells with lentiviral vector particles

All transduction experiments were performed using DMEM with 1 % FCS. Transduction of cells with luciferase-encoding lentiviral vectors for luciferase assays was performed by seeding 6000 HEK 293 T cells per well in white CELLSTAR 384-well microtiter plates (Greiner Bio-One, Frickenhausen, Germany) in a volume of 20 μl DMEM using a MultiFlo Microplate Dispenser (BioTek, Bad Friedrichshall, Germany) [12] and incubating for 24 h at 37 °C. Suramin (Sigma, Darmstadt) or rabbit sera (IBT Bioservices, Gaithersburg, MD, USA), were serially diluted with DMEM until the concentrations were twice those required for the experiment, mixed 1:1 with vector particles (either VSV-G, EBOV GP or CHIKV pseudotyped, produced with pCSII-Luc), and incubated in 96-U-well plates (Thermo Scientific, Rockford, IL, USA) at 4 °C for 1 h. The vector particle mixtures were subsequently added to the cells in the 384-well plates using a Matrix Equalizer Multichannel Electronic Pipette (Thermo Scientific). From every dilution/well in the 96-well plates, 20 μl were transferred to three wells each of the 384-well plates (1:2 dilution; triplicate assay). After 16 h incubation, 20 μl BriteLite substrate (PerkinElmer, Rodgau, Germany) were added to each well using the MultiFlo Microplate Dispenser (BioTek). Following an incubation of 5 min at room temperature, the luciferase signal was detected with a PHERAstar FS microplate reader (BMG LABTECH, Ortenberg, Germany).

### Cytotoxicity assay

For the cytotoxicity test, 4 × 10^4^ 293 T cells per well in a 96-well plate were seeded and incubated with suramin at the same concentrations used in the neutralization assays. Puromycin (2 mg/ml) was used as a positive control for cell killing, while DMEM in the concentration used during the neutralization assay served as a negative control. After incubation for 48 h, the MTT assay was carried out according to the manufacturer’s instructions (Merck Millipore, Darmstadt).

### Zaire Ebola virus infections

The Mayinga strain of *Zaire Ebola virus* (EBOV) (GenBank accession number AF 086833) was used for infections. The virus was propagated in VeroE6 cells and titrated by 50 % tissue culture infectious dose (TCID_50_) assays. EBOV was preincubated at a multiplicity of infection of 0.1 with different concentrations of suramin (4.6875–300 μg/ml in DMEM containing no FCS) at 37 °C for 30 min. Huh7 cells were then infected with ZEBOV and suramin for 1 h at 37 °C. The inoculum was discarded and the cells were supplied with DMEM (5 % FCS, penicillin, streptomycin) containing different concentration of suramin. Supernatants of cells were collected 48 h post infection and their virus titers were determined by TCID_50_ analysis. All work with wild-type EBOV was performed in the biosafety level 4 (BSL4) facility of Philipps University, Marburg.

### TCID_50_ analysis of Ebola virus

VeroE6 cells were cultured in 96-well plates to 50 % confluence and infected with 10-fold serial dilutions of supernatants from infected cells (4 replicates). At 7 days post infection (p.i.), when the cytopathic effect had stabilized, cells were analyzed by light microscopy. The TCID_50_/ml was calculated using the Spearman-Kärber method [32].

### CHIKV and VSV infection

The recombinant CHIKV-luci contains the luciferase gene within the CHIKV nsP3 (non-structural protein 3) [33]. The virus was generated by in vitro-transcription of the plasmid pCHIKV-luci after *Not*I linearization, as described previously [34], followed by transfection of the RNA into BHK-21 cells using Lipofectamine® 2000 (Life Technologies). Supernatants containing virus were harvested 48 h later and the virus was amplified on BHK-21 cells. This replicating luciferase-tagged CHIKV was used to infect 293 T cells at a low MOI of 0.06 and viral replication could be simply detected via luciferase assays. Analogously, VSV infection of target cells was assessed with a VSV encoding luciferase also at a low MOI of 0.2 [35].

### Statistical data analysis

Mean values and standard deviation (SD) were analyzed using Microsoft Excel. Half maximal inhibitory concentration (IC_50_) was calculated using Prism (GraphPad Software).

## Results

### Analysis of suramin’s toxicity

**Fig. 1:**
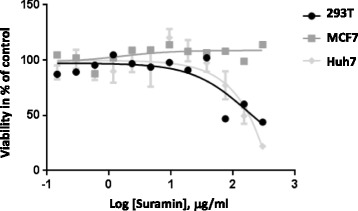
Cytotoxicity of suramin treatment. Cytotoxicity of suramin was analyzed by incubation of MCF7, Huh7 and 293 T cells with suramin for 16 h and conducting a MTT assay by following the manufacturer’s instructions (Merck Millipore, Darmstadt). The graph indicates the amount of viable cells as % of the untreated control and values represent the mean data of two independent experiments done in duplicate

Although both viruses enter cells by different pathways, CHIKV by receptor-mediated endocytosis and a pH-dependent fusion step and EBOV by a combination of attachment and receptor molecules in the endosomes, both viruses attach to cells via GAGs. Consequently suramin, a competitive inhibitor of GAGs should have anti-viral activity. To exclude that the inhibitory effect of suramin is due to toxicity of the compound, the cytotoxicity of suramin was analyzed by incubating different cell lines with suramin for 16 h and performing a MTT assay (Fig. 1). Suramin displayed only negligible toxicity towards MCF7 however affected Huh7 and 293 T cell viability starting at a concentration of 50 μg/ml (Fig. 1). The CC_50_ values were 211 μg/ml for 293 T cells and 182 μg/ml for Huh7 cells. CC_50_ values for MCF7 cells were indeterminable. Therefore effects of suramin at higher concentrations might result from general cell toxicity.

### Suramin inhibits CHIKV and EBOV pseudotyped vector transductions

**Fig. 2:**
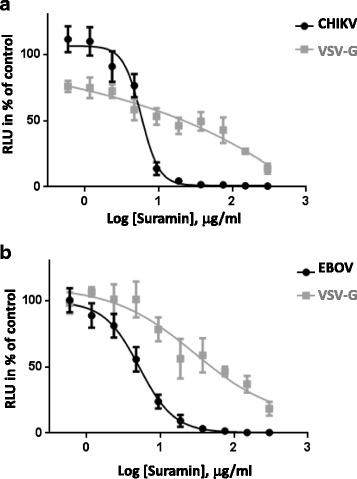
Neutralization assay with suramin. Suramin dissolved in water was serially diluted, incubated with EBOV GP or VSV-G pseudotyped vector particles (**a**) or CHIKV and VSV-G pseudotyped vector particles (**b**) and the mixtures were used for transduction of HEK 293 T cells. Neutralizing activity was determined by detection of relative luciferase units (RLUs). The data represent a typical assay with the mean values of triplicates

CHIKV and EBOV cell entry are GAG-dependent [15, 16]. The market-authorized drug suramin has been described as a competitive inhibitor of GAGs [36, 37] and thus was tested for its inhibitory activity towards CHIKV and EBOV-pseudotyped vectors. Consequently, transduction of 293 T target cells was performed in the presence of increasing amounts of suramin. VSV-G pseudotyped lentiviral vectors were used as a control to exclude effects on the lentiviral background of the vector system. Figure 2 shows that CHIKV (Fig. 2a) and EBOV GP-mediated transduction (Fig. 2b) was effectively inhibited by suramin with a mean half minimal inhibitory concentration (IC_50_) of 4,11 ± 0,74 μg/ml for CHIKV and 8,47 ± 3,21 μg/ml for EBOV. VSV-G-mediated gene transfer was less affected by the presence of suramin (IC_50_ 27,37 ± 11,82) and only high suramin concentrations diminished VSV-G mediated transduction (Fig. 2a, b). This implies that suramin is not toxic at the inhibitory concentrations and only partially inhibits VSV-G-mediated entry, but is able to specifically and fully block CHIKV and EBOV GP-mediated entry.

### Suramin blocks CHIKV infection at early time points

**Fig. 3:**
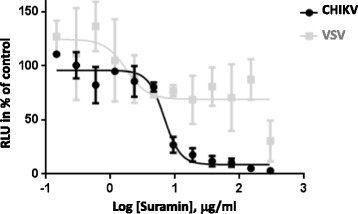
Suramin inhibits CHIKV infection. Suramin was serially diluted, incubated with CHIKV-luci or VSV-luci for 30 min and added to 293 T cells. Its neutralizing activity was detected after 6 h of incubation as relative luciferase activities. The luciferase activity is shown as a percentage, relative to the untreated control. The data show a representative experiment carried out in triplicate

The inhibitory effect of suramin on CHIKV-pseudotyped vector particles was confirmed with wild-type CHIKV. HEK 293 T cells were infected with a replicating luciferase-tagged CHIKV (CHIKV-luci) in the presence of suramin. The recombinant CHIKV-luci contains the luciferase gene within the CHIKV nsP3 (non-structural protein 3) gene [33] and viral replication can be detected via luciferase assays. Figure 3a shows a representative assay with the average values of the experiment done in triplicates. Here, 293 T cells were infected for 6 h with CHIKV-luci in the presence of suramin. Increasing doses of suramin drastically inhibited CHIKV infection of cells (IC_50_ 7,38 μg/ml) (Fig. 3). Effects on VSV infection of target cells were assessed with a VSV encoding luciferase [35]. VSV infection of 293 T cells was only inhibited at high suramin concentrations (Fig. 3).

**Fig. 4:**
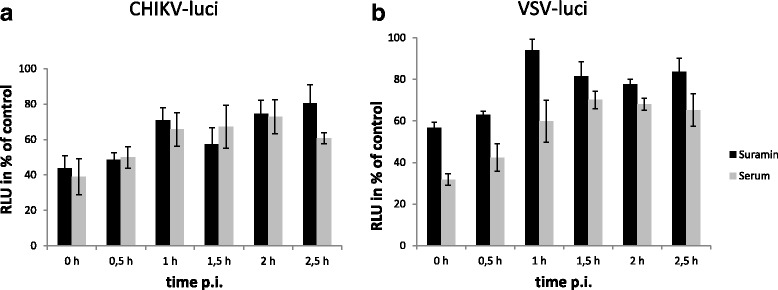
Suramin acts on early steps of CHIKV infections in vitro. HEK 293 T cells were incubated with CHIKV-luci (**a**) or VSV-luci (**b**) and suramin (10 μg/ml). The drug was added during the infection (0 h) and then every 30 min after infection up to 2.5 h after infection. After 6 h, infected cells were detected as relative luciferase activities. CHIKV infection without treatment was set to 100 %

To further analyze the mechanism of viral inhibition by suramin, the drug (10 μg/ml) was added every 30 min for 2.5 h during infection with CHIKV-luci at a low MOI of 0.06 and VSV-luci at a MOI of 0.2 and infection was analyzed after 6 h. Adding the compounds during (0 h) or 30 min after the infection significantly inhibited infection compared to the untreated control. Later addition of the compound had only slight inhibitory effects, however reduced the infectivity to 80 % of the untreated control (Fig. 4a). The same kinetic was also observed when a CHIKV-neutralizing serum was added at the same time points of infection. These data imply that suramin acts on viral entry; however, the influence on later stages of the viral infection cycle might be caused by inhibition of entry of virus released from initially infected cells. In contrast, VSV-luci infection was only inhibited by the neutralizing antibody when added up to 30 min after infection (Fig. 4b). Suramin had only a slight inhibitory effect on VSV when added at all time points of infection.

### Suramin blocks EBOV replication

**Fig. 5:**
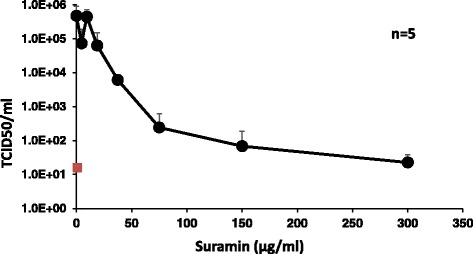
Suramin inhibits EBOV replication in vitro. Different concentrations of suramin (4.875–300 μg/ml) were incubated with EBOV (multiplicity of infection of 0.1) prior to infection of Huh7 cells. Supernatants were collected 48 h post infection and virus titers were determined in Vero cells by TCID_50_ analysis (4 replicates). The data represent the mean values of TCID_50_ titers from 5 independent experiments. The mock value is indicated as a red square

To ascertain the relevance of the data obtained with EBOV GP pseudotyped lentiviral vectors, suramin was added in increasing amounts during a wild-type EBOV infection. The virus, at a multiplicity of infection of 0.1, was preincubated with suramin for 30 min. The mixture was then added to Huh7 target cells, washed off after 1 h, and replaced with medium supplemented with suramin at the previous concentration. Incubation was continued for another 48 h. Subsequently, the viral titer was determined as 50 % tissue culture infectious dose (TCID_50_) values on Vero cells (Fig. 5). Corresponding to the data obtained with EBOV-pseudotyped vectors, there was a distinct inhibition of EBOV replication by suramin with an IC_50_ of 12,58 ± 5,03 μg/ml: even at a low dose, Ebola virus replication was clearly reduced by up to 1-log. These data confirm that suramin is an inhibitor of EBOV with a selectivity index of 14,46.

## Discussion

Suramin has been used as an antihelmintic for the treatment of onchocerciasis (African river blindness) since 1920, and is still the only treatment against the adult worms. Suramin is also used with pentamidine to treat early stages of sleeping sickness (African trypanosomiasis) [38]. However, the mode of action against the parasites is still unknown [39]. Suramin is a market-authorized drug and clinical pharmacology data are available. It is metabolically stable, has a long plasma half-life of 30–60 days in humans, is poorly absorbed from the gastrointestinal tract and 80 % of the drug is excreted renally [39, 40].

For the treatment of onchocerciasis, suramin is administered as a single weekly intravenous injection of 1 g suramin for 6 weeks, which might be sufficient to obtain antiviral effects [41]. The most frequent adverse reactions of suramin-treated patients with onchocerciasis are nausea and vomiting. About 90 % of patients develop a reversible urticarial rash. Kidney damage and exfoliative dermatitis occur less commonly. Suramin is also associated with hepatic and bone marrow toxicity, Stephens-Johnson syndrome, and death. There is a greater than 50 % chance of damage to the adrenal cortex [41]. Other toxic effects of suramin in humans have been documented during clinical trials in cancer patients. Reversible liver toxicity, corneal damage, and adrenal insufficiency have been described frequently [42]. The LD_50_ of suramin in mice is 750 mg/kg [43] after intraperitoneal application and 620 mg/kg following intravenous application [44]. The lowest toxic dose (TD_Lo_) in humans is 46 mg/kg/5 week on an intermittent schedule [45].

Since suramin acts as a competitor of heparin, anticoagulating activity might be expected. However, despite extensive use of suramin, coagulopathy has not been described as a side effect of the treatment. Coagulopathy has only been reported in three female patients receiving suramin as treatment for metastatic adrenocortical carcinoma [46]. For this treatment, a higher suramin dosage of 1.4 g/m^2^/week was used. The study showed that suramin itself did not prolong the clotting time, but rather a suramin-related anticoagulant, which was heterogeneous in the three patients [46].

Here we have shown that suramin’s toxicity is cell line dependent and highest in HEK 293 T and Huh7 cells, whereas MCF7 cells were only marginally affected. Suramin is able to inhibit CHIKV infections. This has been observed before by others [26, 27]. However the mode of action is still unclear. Inhibition of pseudotyped vector transduction and time of drug-addition experiments indicate an effect on viral entry, as observed by Ho et al. [27]. Nevertheless, except the recurrent arthritis, CHIKV fever is, compared to Ebola virus infections, a rather mild disease and the unwanted side effects of suramin might make it inappropriate for the treatment of CHIKV infections. On the other hand, suramin is able to inhibit Ebola virus pseudotyped lentiviral vectors as well as wild-type Ebola virus in vitro and the severity of the disease makes suramin a therapeutic option as long as more specific drugs are not available. VSV-G pseudotyped vector entry was only slightly inhibited by suramin, but never declined to undetectable levels (Fig. 2). This indicates that the inhibition of CHIKV or EBOV entry by suramin uses a different mechanism to that of VSV. Suramin might bind directly to the cell attachment site of the GPs, similar like soluble GAGs and thereby directly compete with cell binding.

Recently, large screens of Food and Drug Administration (FDA)-approved drugs were performed with EBOV VLPs and identified 53 drugs that are able to inhibit EBOV infections [47]. Microtubule inhibitors were the most potent Ebola virus entry inhibitors, with IC_50_ values in the low μM range. However, wild-type virus has not yet been used to confirm the drug activity [47]. Others have reported that amiodarone, a multi-ion channel inhibitor and adrenoceptor antagonist, is a potent inhibitor of EBOV cell entry at concentrations reported to be achievable in plasma in humans [48]. All drugs described so far have a broad mode of action and unwanted side effects might be envisioned. Correspondingly, the wide range of toxicities might require improved analogs of suramin. It should, however, be tested in animal models, which can provide indications about the feasibility of using suramin for Ebola treatment. The last EBOV epidemic in Western Africa was the largest in history with over 10,000 confirmed deaths. Earlier outbreaks only caused a few hundred cases. The long time period until the epidemic was under control and the high case fatality rate (about 41 % for the last outbreak) make rapid and effective actions an urgent requirement for future outbreaks. Suramin treatment of EBOV patients might be option for this, as several decades of treatment experience with other pathogens, especially in Africa, are available. Additionally, suramin can be easily produced in high amounts and would be available immediately. The high lethality of Ebola virus infection makes mild side effects of the treatment acceptable as long as there is no better treatment available. Additionally and in parallel, improvements to the drug to reduce unwanted side effects should be attempted.

## Conclusion

Suramin inhibits CHIKV and EBOV infections in vitro. Suramin might have too many unwanted side effects for the treatment of CHIKV infections, however might be acceptable for the treatment of Ebola virus infections as long as there is no better treatment available. However, appropriate animal models have to show in vivo applicability first.
